# Genetic gains in IRRI’s rice salinity breeding and elite panel development as a future breeding resource

**DOI:** 10.1007/s00122-024-04545-9

**Published:** 2024-01-31

**Authors:** Apurva Khanna, Mahender Anumalla, Joie Ramos, Ma Teresa Sta. Cruz, Margaret Catolos, Andres Godwin Sajise, Glenn Gregorio, Shalabh Dixit, Jauhar Ali, Md. Rafiqul Islam, Vikas Kumar Singh, Md. Akhlasur Rahman, Hasina Khatun, Daniel Joseph Pisano, Sankalp Bhosale, Waseem Hussain

**Affiliations:** 1 Rice Breeding Innovation Platform, International Rice Research Institute (IRRI), 4031 Los Baños, Laguna, Philippines; 2 Southeast Asian Regional Center for Graduate Study and Research in Agriculture (SEARCA) and University of Philippines, 4031 Los Baños, Laguna, Philippines; 3 Plant Breeding Division, Bangladesh Rice Research Institute (BRRI), Gazipur, 1701 Bangladesh; 4 IRRI South Asia Regional Center (IRRI-SA Hub), Hyderabad, Telangana 502324 India

## Abstract

**Key message:**

Estimating genetic gains and formulating a future salinity elite breeding panel for rice pave the way for developing better high-yielding salinity tolerant lines with enhanced genetic gains.

**Abstract:**

Genetic gain is a crucial parameter to check the breeding program's success and help optimize future breeding strategies for enhanced genetic gains. To estimate the genetic gains in IRRI’s salinity breeding program and identify the best genotypes based on high breeding values for grain yield (kg/ha), we analyzed the historical data from the trials conducted in the IRRI, Philippines and Bangladesh. A two-stage mixed-model approach accounting for experimental design factors and a relationship matrix was fitted to obtain the breeding values for grain yield and estimate genetic trends. A positive genetic trend of 0.1% per annum with a yield advantage of 1.52 kg/ha was observed in IRRI, Philippines. In Bangladesh, we observed a genetic gain of 0.31% per annum with a yield advantage of 14.02 kg/ha. In the released varieties, we observed a genetic gain of 0.12% per annum with a 2.2 kg/ha/year yield advantage in the IRRI, Philippines. For the Bangladesh dataset, a genetic gain of 0.14% per annum with a yield advantage of 5.9 kg/ha/year was observed in the released varieties. Based on breeding values for grain yield, a core set of the top 145 genotypes with higher breeding values of > 2400 kg/ha in the IRRI, Philippines, and > 3500 kg/ha in Bangladesh with a reliability of > 0.4 were selected to develop the elite breeding panel. Conclusively, a recurrent selection breeding strategy integrated with novel technologies like genomic selection and speed breeding is highly required to achieve higher genetic gains in IRRI’s salinity breeding programs.

**Supplementary Information:**

The online version contains supplementary material available at 10.1007/s00122-024-04545-9.

## Introduction

Rice (*Oryza Sativa L*.) is a major staple food crop, particularly in Asia, Latin America, and Africa. Rice is most sensitive to salts with EC above four dS/m (Singh et al. 2021; Melino and Tester 2023). Millions of hectares of land in South Asia, Southeast Asia, and Africa are adopted for rice cultivation but have lower yields due to salinity stress effects (Smajgl et al. 2015; Melino and Tester 2023). The future rice food security heavily depends on the rapid development of high-yielding salinity tolerant lines with much better adaptation to the changing climatic scenarios. The salinity breeding program at the International Rice Research Institute (IRRI) has been at the forefront of developing salt-tolerant rice varieties utilizing various donor lines and landraces following conventional breeding approaches (Fita et al. 2015; Singh et al. 2021; Melino and Tester 2023). In the last 2–3 decades, immense efforts have been made at IRRI to develop high-yielding salt tolerance rice varieties through conventional and molecular breeding approaches (Gregorio et al. 2002; Negrão et al. 2011; Platten et al. 2013; Fita et al. 2015; Ismail and Horie 2017; Aala and Gregorio 2019; Singh et al. 2021; Melino and Tester 2023; Eckardt et al. 2023). The salinity breeding program at IRRI was further boosted by the STRASA (Stress Tolerant Rice for Africa and South Asia) project launched in the year 2005 and continued up to 2019 (https://strasa.irri.org/varietal-releases/salinity-iron-toxicity) and Green Super Rice (GSR) project (2006–2016). The progression of these projects led to the identification of new donor lines for vegetative and reproductive stage tolerance and the dissemination of more than 50 varieties for cultivation in saline coastal, saline, and irrigated areas/ecosystems (https://strasa.irri.org/varietal-releases/salinity-iron-toxicity; Ali et al. 2017; Singh et al. 2021). The varieties developed through these projects offer great potential for cultivating them in saline environments to increase rice production.

The South and Southeast Asian coastal regions, which account for 65% of global rice production, are heavily affected by the increased salt-water intrusions as a direct consequence of climate change (Radanielson et al. 2018). The salinity level is expected to increase, significantly impacting rice production in saline ecosystems (Liu et al. 2020; Rawat et al. 2022). In 2050, the human population will reach 10 billion, and the demand for rice production will increase by 87% (Solis et al. 2020; Rawat et al. 2022). Due to the limited resources and less availability of land in the future, meeting the future rice demands is a daunting challenge. Moreover, future rice production will only be met with heavy reliance on irrigation water (Liu et al. 2020). However, dependency on irrigation water for rice production comes with an additional cost of land salinization, and the level of dissolved salts in irrigation water has significantly increased in the past 20 years (Liu et al. 2020). Thus, global rice food security mainly depends on plant breeders to develop high-yielding, salinity-tolerant lines with broader adaptation. To develop improved and widely adopted salinity-tolerant lines, evaluating the progress of the existing salinity breeding program is essential. This evaluation will provide valuable insights into the program's current state and help identify areas for improvement and opportunities to enhance genetic gains.

Genetic gain is an important parameter to check the progress of the breeding program and measure its efficiency. The breeding program's achieved rate of genetic gain will immensely help guide future breeding strategies and help allocate resources and rapid development of varieties for enhanced genetic gains. Genetic gains under salinity environments at the global level in rice have never been estimated. Thus, this study was undertaken to accomplish two primary objectives: (i) estimating the genetic trends in the IRRI’s salinity breeding program using the trial data conducted at IRRI, Philippines, and Bangladesh, and (ii) identifying top-performing genotypes based on high grain yield breeding values as future breeding resources.

## Materials and methods

### Breeding materials and experimental details

For this work, the historical datasets from salinity breeding trials conducted at various locations in IRRI, Philippines, from 2008 to 2019 (12 years) and Bangladesh from 2005 to 2014 (10 years) were used. The major traits of focus were grain yield (kg/ha) and days to flowering. The Bangladesh trials were undertaken in the districts Satkhira (22.7185° N, 89.0705° E), Ghazipur (25.5878° N, 83.5783° E), and Rajshahi (24.3745° N, 88.6042° E). The trials were organized twice a year, in two season’s dry and wet seasons in the Philippines and Aman and Boro in Bangladesh. The genotypes were staggered based on their maturity groups: early, medium, and late to synchronize appropriate stress imposition. The genotypes were screened for tolerance to salinity stress across trials starting tillering onwards or at the reproductive stage. The genotypes were planted in customized saline microplots for imposing salinity stress, and the standard protocol was used to screen for the salinity stress. The experimental designs in the trials conducted in the Philippines varied across years from randomized complete block design (RCBD), row-column design, augmented RCBD, and alpha lattice; however, all the trials conducted in Bangladesh were organized in RCBD.

### Pre-processing and quality check of the data

The breeding values were estimated yearly, taking season and location combinations as a single trial or environment. The historical datasets retrieved were subjected to pre-processing and quality checks to ensure high-quality trials and phenotypes are retained for the downstream analysis and estimating the breeding values and genetic gains. The data pre-processing was done per the procedure detailed in the manuscripts (Hussain et al. 2022; Khanna et al. 2022a). Trials with unexpected phenotypic values, high missing data points (> 20%), missing replications, and/or design errors were filtered. After filtering, the trials were subjected to quality checks by removing the extreme data points and outliers using the Bonferroni-Holm test for studentized residuals (Bernal-Vasquez et al. 2016; Philipp et al. 2018, 2019). After pre-processing and quality check, the dataset consisted of 86 trials with 16,251 phenotypic data points with 4993 unique genotypes from IRRI, Philippines datasets. For Bangladesh, 110 trials possessing 3097 data points with 600 unique genotypes were retained. The details of the trials conducted across the two countries are outlined in Supplementary Table 1.

### Retrieval of pedigrees and crossing strategies

The pedigree data consisting of the parent's and grandparent's information on the 4,993 genotypes was utilized for substituting the pedigree-based relationship matrix in the Philippines dataset only. Pedigree information was not available for the datasets from Bangladesh. The information of grandparents up to ten generations, along with the crossing strategy employed for each genotype, was retrieved from the state-of-the-art repository, B4R (Breeding 4 Results; https://b4r.irri.org) and IRRI genealogy management system (McLaren et al. 2005; Collard et al. 2019) with their customized R scripts. The genotypes were bred across the years employing various breeding strategies, single, double, three-way, complex crosses, and backcrosses based on their breeding objectives. The pedigree information of the genotypes for Bangladesh was unavailable, and BLUPs for grain yield were extracted instead of breeding values.

### Statistical modeling

Due to different experimental designs across the trials and to account for the specific experimental design factors, the two-stage approach of mixed-model analysis was used (Smith et al. 2005; Piepho et al. 2008, 2012). The two-stage approach also reduces the time and computational burden of analyzing huge datasets (Smith et al. 2005). In the first stage, adjusted means or best linear unbiased estimates (BLUEs) per year for each genotype were extracted for grain yield. The mixed model consists of genotypes as fixed effects with replications and seasons as random effects. Days to flowering (DTF) was used as a covariate in the model to reduce error due to the difference in the flowering synchronization and ensure the selections for best genotypes would be across different maturity groups. The strategy of covariance adjustment of DTF would significantly reduce variance due to differentiation in flowering time among the genotypes in the analysis (Moreno-Amores et al. 2020; Juma et al. 2021; Khanna et al. 2022b, a). The baseline model used in the first stage of analysis is given below:

1 $$y_{ij} = \mu + g_{i} + s_{j} .... + \varepsilon_{ijk }$$

where ${y}_{ij}$ represents the response variable grain yield (kg/ha) for ith observation, *μ* is the overall mean, *g*_*i*_ is the fixed effect of ith genotype, *s*_*j*_ is the fixed effect of jth season, and ${\varepsilon }_{ijk}$ is the residual error. The random effects were independently and identically distributed (IID). The … in the model (1) denotes the blocking factors block, replications, row, column, and DTF as covariate. These terms were included in the model based on the experimental design. For trials with a row-column design, the factors were row and column, for trials with RCBD or augmented RCBD, the factor was replicate and block, for those with an alpha-lattice design the possible factors were replications, and blocks nested within replications.

In the second stage, the BLUEs estimated from the first stage were weighted and used as a response variable (Damesa et al. 2017; Hussain et al. 2022; Khanna et al. 2022a). The weights were estimated by calculating the inverse of the squared standard errors (Möhring and Piepho 2009), which minimized the heterogeneous error variance. In this stage, a relationship matrix based on the pedigrees was fitted to account for the genetic covariances among the genotypes for reliable estimates of breeding values. The same model was used for the Bangladesh dataset without fitting the pedigree matrix to extract the BLUPs. The model fitted in the second stage is as follows:

2 $$y_{ij} = \mu + g_{i} + e_{j} + \varepsilon_{ij}$$

where ${y}_{ij}$ is the BLUE values weighted by the standard errors for *i*th observation in *j*th year, μ is the overall mean, ${g}_{i}$ is the breeding value of *i*th genotype with g_i_ ∼ N (0, Aσ^2^_g_) where σ^2^_g_ is the genetic variance and A is the additive genetic pedigree relationship matrix, ${y}_{j}$ is the fixed effect of *j*th environment, and ${\varepsilon }_{ij}$ is the residual error, with ${\varepsilon }_{ij}$ ∼ N (0, Rσ^2^_ε_), where R is the identity error covariance matrix and σ^2^_ε_ is the error variance. The reliability values (Isik et al. 2017) of the breeding values with respect to each genotype were calculated using the equation mentioned below:

3 $$r = 1 - \frac{PEV}{{\sigma_{g}^{2} }}$$

where PEV is the prediction error variance for each breeding value and σ^2^_g_ is the genetic variance.

Heritability for the yield was estimated using the method suggested by (Cullis et al. 2006) and (Piepho and Möhring 2007) using model 1 with modifications. In model 1, the season effect (*s*_*j*_) was removed as heritability was estimated per season, and genotypes were treated as random. This approach is useful when the data are highly unbalanced, with uncommon genotypes screened across the years and seasons. The following equation was used to calculate the heritability for trials per year:

4 $$H^{2} = 1 - \frac{{\overline{V}_{BLUP} }}{{2\sigma_{g}^{2} }}$$

All the analyses were done using the ASReml-R package (Butler et al. 2018) in the R software (R Core Team 2023). The pedigree-based relationship matrix (A-matrix) was constructed using the R package AGHMatrix (Amadeu et al. 2016).

Various approaches and methodology have been used to estimate the genetic gains with the historical data (de la Vega et al. 2007; Mackay et al. 2011; Sharma et al. 2012; Piepho et al. 2014; Laidig et al. 2014, 2017; Morais Júnior et al. 2015; Streck et al. 2018; Hoyos-Villegas et al. 2019; Muralidharan et al. 2019; Lozada and Carter 2019; Kumar et al. 2021; Prasanna et al. 2022; Rahman et al. 2023). The methodology used in this study was selected based on the unbalanced structure of the data, availability of pedigree information, and poor connectivity over consecutive years. A similar methodology has been used by (Juma et al. 2021; Khanna et al. 2022a) in estimating the genetic gains with historical data by leveraging the pedigree-derived relationship matrix.

### Estimation of the genetic trends

For the IRRI, Philippines data, the genetic gains were estimated by regressing each genotype's breeding values over the year of origin or the year when the cross was attempted for each genotype. The year of origin for each genotype record was extracted using the customized R scripts from the genealogy management system IRRI (McLaren et al. 2005; Collard et al. 2019). However, in the case of Bangladesh, the genetic trends were estimated by regressing the BLUPs over the year of testing for each genotype. To estimate the gains only with released varieties, a similar strategy was followed by regressing each genotype’s breeding values or BLUPs over the year of release for each country. Additionally, genetic gain trends were plotted using the non-linear approach of *loess* (local weighted regression) to check the short-term and long-term genetic trends in the salinity breeding program at IRRI, Philippines, and Bangladesh datasets.

### Formulation of elite breeding panel

Breeding values or BLUPs for grain yield obtained from the second-stage analysis were used to formulate the salinity breeding panel as a future genetic resource. Based on the higher breeding values of > 2300 kg/h in the Philippines and BLUPs > 3550 kg/h in Bangladesh and reliability of > 0.4, 145 genotypes were selected as a part of the breeding panel. For the IRRI, Philippines dataset, genetic similarity between the selected lines and in comparison with the whole historical line collection was assessed using the relationship matrix based on pedigrees. The diversity and similarity of the lines over the complete set of 4993 genotypes from the historical salinity dataset were visualized through the biplot graph. The variables for the biplot were obtained through the principal component analysis (PCA) performed using the function *princomp* in R software on the A-matrix or pedigree matrix.

### Genetic trends of released lines

From the IRRI, Philippines data, 17 IRRI-released saline-tolerant varieties were utilized to estimate the genetic gains. Similarly, the gains were estimated using 12 released salinity-tolerant varieties in Bangladesh. The breeding values from the Philippine dataset and BLUPs from Bangladesh data were regressed to their year of release for estimating the genetic gains. Also, to further understand the breeding program's growth, recently nominated 25 IRRI varieties across nine countries for the year 2021–22 were compared for their breeding values.

Superior-performing 12 genotypes comprising seven nominated varieties and four selected varieties from the historical core panel possessing higher breeding values and salinity tolerance were tested for stability using their grain yield performances in 5 environments, viz*.* in the year 2018 at IRRI, Philippines, and Ajuy Iloilo during the wet season: in the year 2019 at IRRI, Philippines, during dry and wet seasons and at Ajuy Iloilo during the wet season. Stability analysis was performed using R software's GGE Biplot GUI package (Frutos et al. 2014). The percentages of GGE explained by the top two PC axes were estimated for ranking genotypes based on their relative performance and ranking genotypes relative to the ideal genotype. The analysis was based on a Tester-centered (G + GE) table without scaling and with row metric preserving.

## Results

### Description of historical salinity datasets

**Fig. 1:**
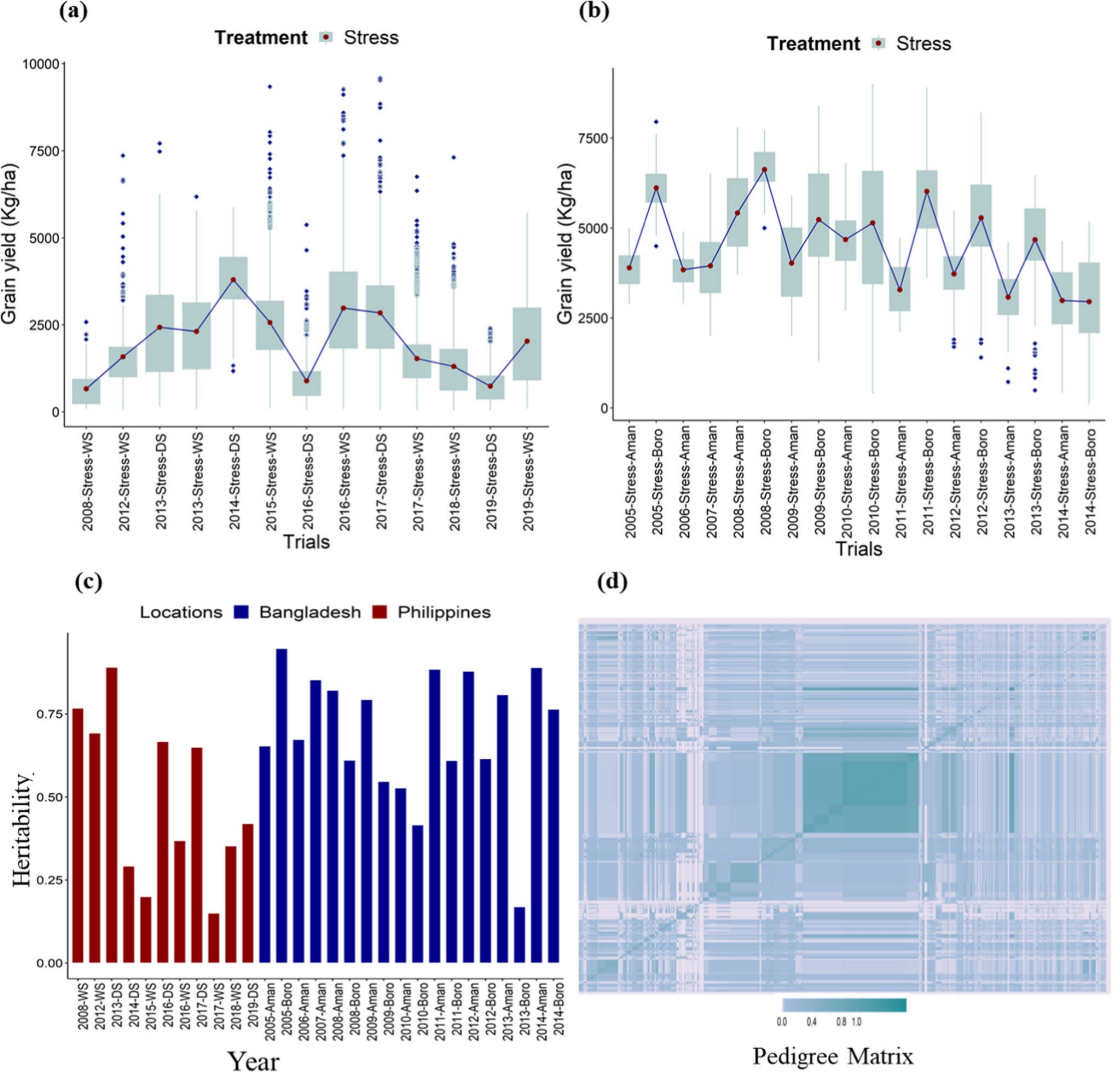
Boxplots depicting the grain yield (kg/ha) across breeding trials **a** 2008–2019 at the Philippines in the dry and wet seasons, **b** 2005–2014 in Bangladesh during aman and boro seasons. **C** The bar plot shows the heritability of the trials per season **d** Heat map based on relationship matrix of 4,993 unique genotypes bred across 12 years at IRRI, Philippines. As the color scale progresses from light blue through sea green in the off-diagonals to dark cyan in the diagonals, the genetic similarity increases among the genotypes, scaling 0.4 to 0.8 to 1

A high difference in the mean values for grain yield (kg/ha) was observed in the Philippine and Bangladesh datasets (Fig. 1 a, b). For the DTF in the Philippines dataset, two maturity categories of early and medium were found, with 98% (DTF: 66–109 days) of genotypes falling under early and 2% (DTF: 110–124 days) genotypes under medium maturity groups. However, in the Bangladesh dataset, the genotypes were found to fit into all three maturity groups, with 66% of genotypes possessing DTF values between 70 and 109 days, 27% of genotypes having DTF ranges between 110 and 124 days, and 7% of the genotypes were the late category with DTF ranges between 124 and 135 days, respectively.

The heritability based on BLUP differences for grain yield was estimated for each year. The heritability estimates ranged between 0.20 and 0.89 for the trials from 2008 to 2019 in the Philippines dataset (Fig. 1c). Similarly, in the historical dataset from Bangladesh, the heritability ranges were between 0.17 and 0.95 (Fig. 1c). The heritability values were very low in few of the seasons due to the environmental/season/year influence of genotypes to the salinity stress conditions, which would, in turn, affect the grain yields (Rauf et al. 2012). This could be due to different stress levels and environmental conditions across the years, as the priority objective of the stress trials would be to impart higher salinity stress for identifying tolerant genotypes.

### Historical data connectivity

**Fig. 2:**
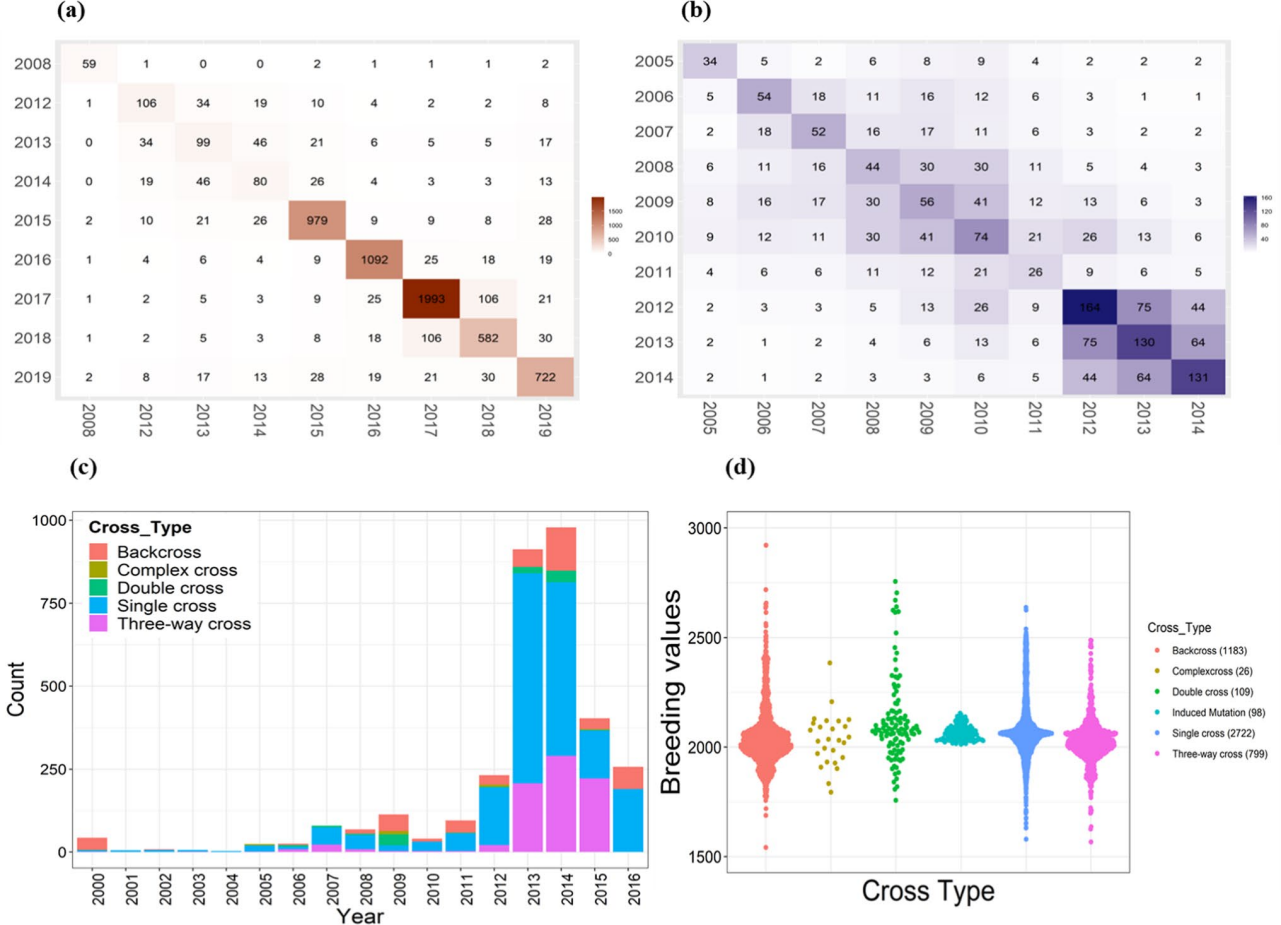
**a** Connectivity of all the unique genotypes in the Philippines dataset. b Bangladesh dataset across years. The numbers in each box represent common genotypes between each year combination. **c** The bar plot depicts the varying breeding strategies and crossing schemes implemented at IRRI, Philippines, from 2000 to 2016. As the figure depicts, single and backcrosses were initiated in the initial years, which were added by double, three-way complex crosses in the later years in the breeding program. **d** The boxplot depicts the range of each breeding strategy in terms of breeding values. However, the ranges slightly differ in each case; the backcrosses possessed a wider range, followed by single and three-way crosses. The numbers of each cross combination formulated across years have also been depicted in the brackets beside each cross-type. The maximum were single cross combinations followed by backcrosses

The connectivity in the historical dataset is a major parameter affecting breeding values and genetic gains estimates. The historical data used in this study have very low connectivity as new lines are tested every season and year, and the new lines were advanced and re-tested only a few times. To establish the connectivity among the datasets and ensure reliable estimates of the breeding values, a relationship matrix (Fig. 1d) based on pedigrees was incorporated in the second stage of mixed-model analysis. The establishment of the connectivity among the unbalanced historical datasets using the pedigree relationship matrix has been shown in the drought data (Khanna et al. 2022b, a). Additionally, the suitable connectivity in the current datasets can be attributed to the common saline tolerant (FL478, Pokkali-8558) and susceptible checks and varieties (IR29, IRRI 104, IRRI 165, etc.) used in the breeding trials across seasons and years. Alongside, the connectivity was maintained as the tolerant genotypes/varieties were evaluated in subsequent years to reconfirm their stable tolerance across years (Fig. 2a, b). It was also observed that there was connectivity among the trials across seasons in each year in both countries (Supplementary Fig. 1a, b, c, d).

### Crossing strategy across salinity historical breeding program

It is crucial to decipher the crossing strategy, or the breeding scheme adopted by the breeders during this period and associate it with the genetic gain trends. The crossing strategy used was extracted from the B4R database. From 2000 to 2016, the breeders performed single, double, three-way, complex, and backcrosses (Fig. 2 c, d). It was found that during the initial years, 2000 to 2005, most of the crosses were single and backcrosses, most of which, until 2012, included double, three-way, and complex cross combinations. However, post-2012, the era when IRRI was rephrasing from marker-assisted backcross breeding to complex/multiple crossing strategies for developing various stress-tolerant breeding varieties, the complex crossing strategy was more highlighted along with three-way and backcrosses (Fig. 2c). The unique 4, 993 genotypes from the historical breeding trials dataset of Philippines had a broad background with differing parents and cross combinations and can also be classified into 770 families based on their diverse parental crosses, among which 108 families were derived from backcrosses; 451 from single-cross combinations; 49 from double-cross combinations; 152 from three-way crosses; 13 from complex crosses, two from induced mutations procedures and remaining were landraces; and remaining 5 were accessions and donors from the gene bank. However, no clear association was found between the crossing strategy and change in the genetic gain trends. After 2012, more fluctuations were observed with genetic trends because the breeding program focused on complex/multiple crossing strategies to develop multiple stress-tolerant breeding lines with a limited focus on using population improvement strategy to enhance the yield.

### Estimation of breeding values and genetic trends

**Fig. 3:**
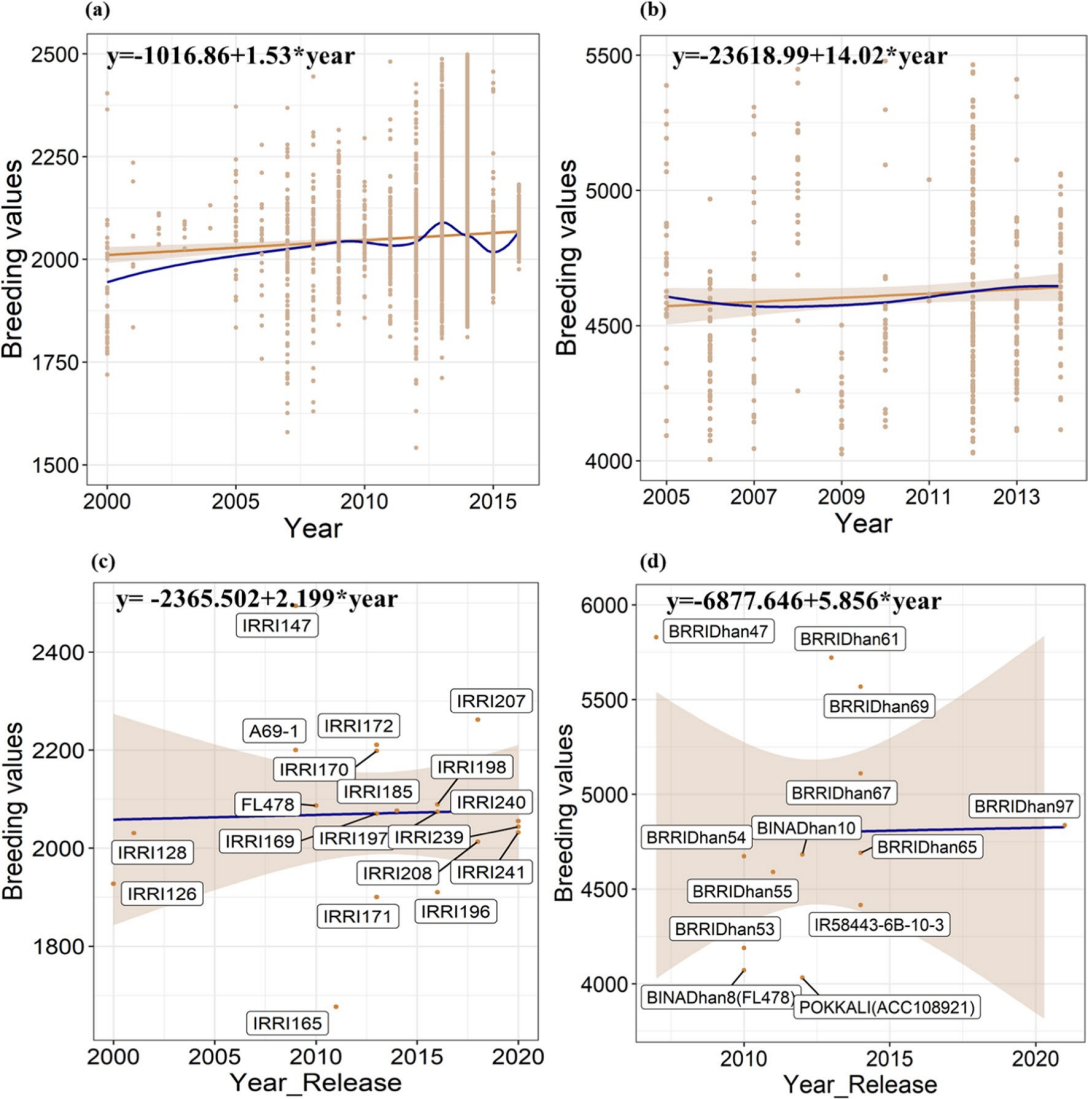
**a**Trends in genetic gain from IRRI’s salinity breeding program comprising 12 years of breeding trials, 2008 to 2019. **b** Trends in genetic gain from salinity breeding program in Bangladesh comprising 10 years of breeding trials, 2005 to 2014. In both (a) and (b), the x-axis depicts the year of origin, and the y-axis portrays the breeding values or BLUPs of the genotypes. The dots represent the breeding values or BLUPs respective to each year. The slope in peru represents the genetic trends using linear regression, and the slope in dark blue portrays the genetic trend using a nonparametric approach using loess regression. **c** The trends in genetic gain for the released varieties across years bred for salinity tolerance at IRRI. **d** The trends in genetic gains for the released varieties across years bred in Bangladesh. The gains were likewise estimated by regressing the grain yield breeding values on the year of origin

The breeding values ranged between 1326.06 and 4720.35 kg/ha in the Philippines dataset and 3587.27–5829.77 kg/ha in the Bangladesh dataset (Supplementary Fig. 2a, b). The genetic gain at IRRI, Philippines, between 2008 and 2019 was 0.1% per annum, with a yield advantage of 1.53 kg/ha/year. The gain was estimated by regressing the year of origin or year of crossing for each of the genotypes spanning from 2000 to 2016, tested in the breeding trials across the years 2008 to 2019 (Fig. 3a). In Bangladesh, the genetic gain was 0.31% per annum with a yield advantage of 14.02 kg/ha per annum (Fig. 3b). Both in IRRI, Philippines, and Bangladesh datasets, linear and nonlinear genetic trends were plotted to check the fluctuations in genetic trends over the years (Fig. 3a, b). The genetic trends were estimated for the released varieties in the Philippines and Bangladesh. The genetic gain was 0.12% per annum with a yield advantage of 2.2 kg/ha/year as estimated for the released varieties at IRRI, Philippines (Fig. 3c). In Bangladesh, the annual gain in grain yield for salinity was 0.14% with a yield advantage of 5.9 kg/ha/year (Fig. 3d).

### Development of an elite breeding panel

**Fig. 4:**
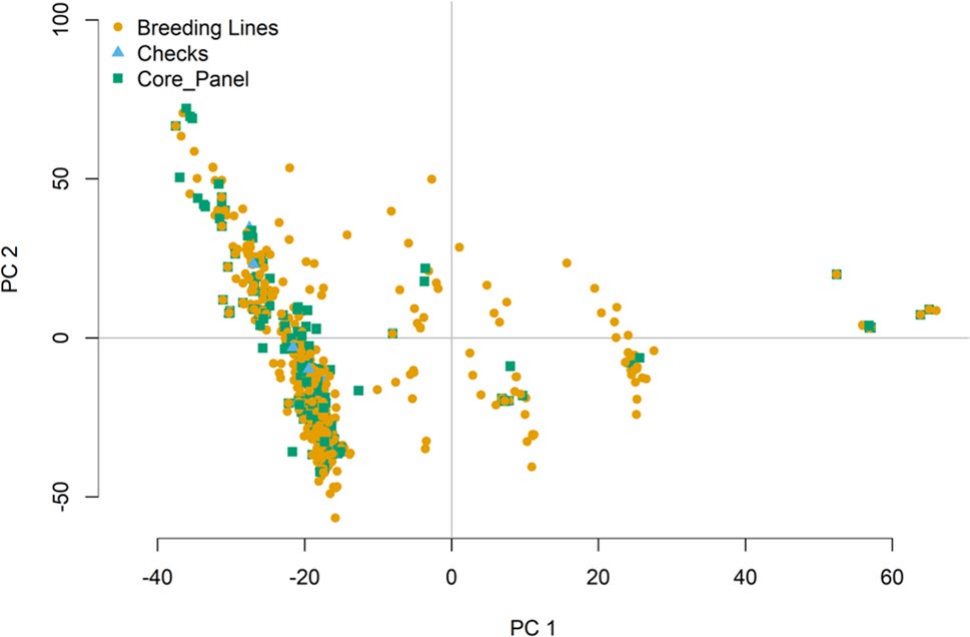
PCA-biplot depicting the complete set of unique breeding lines obtained from the historical breeding trials in IRRI, Philippines. The elite core panel breeding lines selected based on the superior breeding values have been depicted by violet color and checks by blue color. The biplot was obtained and plotted using the first two principal components using the pedigree matrix. The elite core panel lines overlap the complete set of breeding lines and represent the diversity of whole collection as a useful future breeding resource

The genotypes with higher breeding values of > 2400 kg/ha in the Philippines and > 3500 kg/ha in Bangladesh and having reliability > 0.4 were selected for formulating the elite breeding panel. The top 145 genotypes were selected as a future breeding resource for the elite core panel. The criteria for selecting the elite lines, their breeding values, cross combinations, and crossing strategies employed are given in Supplementary Table 2. We also accessed the kinship of the lines from IRRI, Philippines data, using the pedigree relationship matrix (Fig. 4a). The genotypes selected for the elite core panel represent the whole data collection and cover the diversity of the entire collection very well. The genotypes selected for the elite panel not only possess a high breeding value for yield but harbor salinity-tolerant landraces, including Sadri, Pokkali, and Cheriviruppu; elite breeding genotypes like NERICA (New Rice for Africa), which are early maturing (< 100 days); tolerant to major stresses of Africa; AT401, variety can withstand coastal saline environments; zinc-fortified genotypes (IR68144; BR7840-54–3-1); zinc-efficient donor parents (IR55179). The panel additionally possesses genotypes with superior characters, including genotypes with superior yields under DSR conditions (Supplementary Table 3), zinc-efficient genotypes with superior breeding values, iron toxicity tolerant genotypes, and coastal and acid saline-tolerant genotypes (Supplementary Table 2).

### Comparison of breeding values in IRRI-released, nominated, and core panel lines

The breeding values for the IRRI-released varieties were compared with the saline-tolerant checks and varieties for estimating the gains obtained in the released varieties. The comparative analysis would help to comprehend the genetic progress and identify superior genotypes across the years. The breeding values of the released varieties were superior to the popular checks for both countries. In the Philippines dataset, the released global salinity varieties IRRI 147, IRRI 207 and IRRI 172, IRRI 170, and IRRI 198 depicted superior breeding values of 2493.84 kg/ha, 2261.63 kg/ha, 2210.60 kg/ha, 2198.40 kg/ha, and 2089.28 compared to other salinity-tolerant varieties and popular checks A69-1 and FL478 with the breeding value of 2200 kg/ha and 2086.74 kg/ha, respectively. Among these superior-performing varieties, IRRI 207 and IRRI 198 were recently released in the years 2018 and 2016 (Fig. 2c). In the Bangladesh dataset, all the genotypes included for estimating the gain were superior performing to salinity tolerant check FL478. Among all, BRRI Dhan 47, BRRI Dhan 61, BRRI Dhan 69, and BRRI Dhan 67 depicted superior breeding values of 5829.77 kg/ha, 5720.48 kg/ha, 5568.19 kg/ha, and 5110.41 kg/ha, respectively, to the salinity-tolerant check FL478 (BINA Dhan 8) and Pokkali with the breeding values of 4071.16 kg/ha and 4021.65 kg/ha, respectively (Fig. 2d). Among these varieties BRRI Dhan 61, BRRI Dhan 69 and BRRI Dhan 67 were released in the years 2013 and 2014, respectively.

Alongside the released varieties across years, 25 IRRI-bred varieties nominated for eight countries in 2021–22 were also compared for their breeding values. The breeding values of these varieties ranged between 2025.80 and 2920.67 kg/h (Supplementary Fig. 3). Overall, IR121094-B-B-AJY3-2-B (IR18T1021) nominated for release in Thailand depicted the highest breeding value of 2920.67 kg/h followed by IR121188-28–1-CMU2-2-B (IR18T1015), nominated for release in the Philippines with a breeding value of 2718.15 kg/h. Interestingly, the latter possesses higher breeding value compared with the till date-released varieties utilized for estimating gains for 2008–2019 and among other nominated varieties for the year 2021–22 for the Philippines. Additionally, IR121188-28–1-CMU2-2-B was also found to be the most stable variety based on the relative performance when ranked among the other varieties, including IRRI 147, which possessed the highest breeding value among the date-released varieties for the Philippines, and was found the most preferred variety residing along relative to the ideal genotype as shown by the arrow in the Supplementary Fig. 4.

Further, succeeding IR117676-318–1-1–1 has been nominated for Sri Lanka, which possesses a breeding value of 2640.39 kg/h and ranked fourth for stability among the tested varieties, followed by IR112462-B-25–2-1–1 (IR16T1631) nominated for Bangladesh and Lao PDR with a breeding value of 2625.29 kg/h. IR16T1009, nominated for Thailand, also depicted a breeding value of 2514.73 kg/h. This was followed by IR63307-4B-4–3, nominated for Indonesia, Vietnam, and Sri Lanka with a breeding value of 2493.843 kg/h, bred using a soma clonal variant of Pokkali. Another variety, IR117839-22–15-B-CMU10-1-B, nominated for release in the Philippines and Vietnam, possessed a breeding value of 2479.29 kg/h and was the second most stable genotype among the tested varieties in the five environments.

In the selected panel, 8% of the genotypes comprise IRRI-bred varieties that can withstand salinity stress in hand with superior performance under DSR conditions with yields ranging between 2195 and 4758 kg/h (Supplementary Table 3). Regarding including the genotypes from current year nominations, 12% of the panel comprises recently nominated varieties with the highest breeding values. All eight superior breeding value harboring genotypes of the nominations, viz. IR121094-B-B-AJY3-2-B (2920.67), IR121188-28–1-CMU2-2-B (2718.15), IR117676-318–1-1–1 (2640.39), IR112462-B-25–2-1–1 (2625.29), IR16T1009 (2514.73), IR63307-4B-4–3 (2493.843), IR117839-22–15-B-CMU10-1-B (2479.29), IR117749-B-B-CMU6-1-B (2379.65), were part of the core breeding panel (Supplementary Table 2). Another 3% of the panel formulates zinc and iron bio-fortified genotypes with superior breeding values of > 2400 kg/ha for being future-ready for upscaling salinity tolerance and additional characteristics.

## Discussion

We demonstrated the genetic trends in IRRI’s rice salinity breeding program by leveraging historical data and pedigree information. Besides genetic gain estimates, top-performing genotypes based on high breeding values for grain yield were also identified as a future elite breeding resource. Availability of the pedigree information from the IRRI data was crucial to fit and use in the second stage of analysis for reliable estimation of the breeding values to help in the identification of accurate genotypes for the development of the elite panel and accurate estimation of genetic gains (Rutkoski 2019). As these lines have already been bred under saline environments, they are not only tolerant to salinity but possess high breeding values for yield, making them readily available genetic resources for the population improvement-based breeding strategy, further enhancing the genetic gains in the salinity breeding program.

### Genetic gain estimations

One of the main goals of the breeding program is to sustain the genetic gains while maintaining genetic diversity (Cowling et al. 2017; Lehermeier et al. 2017; Gorjanc et al. 2018; Allier et al. 2020). The genetic gain pertains to the success and growth of the breeding program through an increase in the mean performance of the population over the years of selection (Ramstein et al. 2019, Rutkoski et al. Rutkoski et al. 2019b). The current work depicted positive genetic gains in the salinity breeding program, with an improvement rate of 0.33% in Bangladesh and 0.13% in the IRRI, Philippines. However, the observed genetic gains were below the expected rate of 1.5% or above to meet the rice consumption demand (Li et al. 2018). Recently, a study from Bangladesh reported very low genetic gains for grain yield in the rice varieties released between 1970 and 2020 (Rahman et al. 2023). Low genetic gains of < 0.5% have been reported in the irrigated and drought rice breeding programs of IRRI (Kumar et al. 2021; Juma et al. 2021; Khanna et al. 2022a). Comparatively, higher rates of genetic gain of around 1% have also been reported in the different rice breeding programs (Peng et al. 2000; Peng and Khushg 2003; Tabien et al. 2008; Breseghello et al. 2011; Zhu et al. 2016).

To achieve the required rates of genetic gain in the IRRI’s rice salinity breeding program, a major tweaking in the breeder’s equation through modernization and optimization is highly required (Cobb et al. 2019; Merrick et al. 2022). For example, the current salinity breeding has been strictly aligned to a recurrent selection scheme with an elite x elite crossing strategy to maintain the genetic variance and sustain the genetic gains (Breseghello et al. 2009; Júnior et al. 2017; Allier et al. 2019a; Cobb et al. 2019; Dreisigacker et al. 2023a). Cycle time (*t*), a crucial parameter in the genetic gain equation (Araus et al. 2018; Cobb et al. 2019), has been optimized, and cycle time has been reduced to 3 years for quicker recycling of lines, thus speeding the process of improving the mean performance of the population (Baertschi et al. 2021). Selection intensity (*i*) and accuracy (*r*) have been improved by integrating the genomic selection in the breeding program, selecting and advancing the lines based on the breeding values rather than phenotypic BLUPs (Heffner et al. 2009; Jannink et al. 2010; Desta and Ortiz 2014; Crossa et al. 2017; Chung and Liao 2020, 2021; Dreisigacker et al. 2023b). Replicated experimental designs with robust analysis based on mixed-model approaches accounting for spatial trends and G x E interactions have been incorporated into the program to improve heritability and selection accuracy (Voss-Fels et al. 2018; Cooper et al. 2020; Xu et al. 2020; Cooper et al. 2021; Xu et al. 2022; Cooper et al. 2023; Nguyen et al. 2023). In the future, the salinity breeding program is targeting to move to a 2-year breeding cycle by integrating genomic selection with speed breeding and early recycling of the genotypes (Jighly et al. 2019). Further, in the future, the breeding program will focus on maintaining the genetic variance to achieve short-term and long-term genetic gains using the optimal parental contribution selection schemes (Gorjanc et al. 2018; Allier et al. 2019a, b; Santantonio and Robbins 2020). A new breeding approach called “connected breeding” has been developed and integrated into the breeding program to run the population improvement and diversification of the elite pool in parallel without decreasing the mean performance of the elite lines (Sanchez et al. 2023). The connected breeding approach promises to enrich the elite gene pool with previously under-utilized salt-tolerant landraces or non-elite lines without directly crossing them to the elite pool. This approach can transfer the additional quantitative genetic variation from the non-elite pool to the elite breeding pool without interrupting the current elite x elite breeding strategy and mean population performance (unpublished). Thus, a well-focused population improvement program with systematic pre-breeding efforts, quick recycling, robust experimental design, mixed-model analysis, characterization of environments, and defining the target product environments (TPE) are the future targets of the breeding program to deliver constant and higher rates of genetic gains.

### Core panel formulation for identifying elite genotypes

A set of high-performance, elite breeding lines with salinity tolerance is highly required to unlock the potential of cultivation in saline soils with enhanced genetic gains. The conventional salinity breeding at IRRI has mainly focused on crossing the non-elite (salinity tolerant traditional donors/landraces) to the high-yielding elite breeding lines to develop the high-yielding elite salinity tolerant lines (Gregorio et al. 2002; Singh et al. 2021; Melino and Tester 2023). Further, since the inception of molecular breeding, the main focus has been on the introgression or pyramiding of salinity-tolerant QTLs in elite backgrounds (Singh et al. 2021). The rice breeders have used different crossing strategies (Fig. 2 a, b, c) single, complex, double, and backcrosses to integrate these QTLs into the elite genetic backgrounds and develop the new breeding lines. Diverse materials, including landraces and donors, have been extensively used to diversify the gene pool and develop climate-resilient salinity varieties (Singh et al. 2021; Yadav et al. 2021; Sandhu et al. 2021). The genotypes used in this study represent the breadth of the diversity of IRRI’s salinity breeding program through which several high-yielding salinity tolerant varieties have been released (Singh et al. 2021; Melino and Tester 2023). This breeding resource represents an essential source of elite genetic variation that can be leveraged to extract the diverse genotypes with high breeding values for grain yield and salinity tolerance as a future genetic resource. To this end, an effort was made to develop the representative set of the elite pool from this historical collection based on high breeding value and genetic divergence. The developed elite pool can be readily used in a rapid, recurrent selection-based breeding strategy to quickly re-cycle the lines for enhanced genetic gains. Recurrent selection is critical to increase the frequency of favorable additive alleles of grain yield and enhance genetic gains (Breseghello et al. 2009; Morais Júnior et al. 2015, 2017, 2018; Grenier et al. 2015; Abdulmalik et al. 2017; Gorjanc et al. 2018; Bijma et al. 2020; Ramasubramanian and Beavis 2020; Juma et al. 2021; Baertschi et al. 2021; Khanna et al. 2022a; Pereira de Castro et al. 2023).

For example, the elite pool identified in this work has released lines like BRRI Dhan 55, BRRI Dhan 47, BRRI Dhan 67, IRRI 185, IRRI 235, and IRRI 147, which showed high breeding values for the grain yield (Fig. 3; Supplementary Table 2). Specifically, the released variety IRRI 147 for the Philippines, also released as BRRI Dhan 47 in Bangladesh, depicted the highest breeding values among the released varieties. The variety harbors a unique characteristic of erect plant architecture as its leaf angle falls between 5 and 20º (BRRI annual report 2018–2019). The erect plant architecture renders higher photosynthetic abilities by impacting the source and sink organs, making it crucial to identify genotypes with superior “ideotypes,” which can significantly enhance yield, productivity, and gains (Chang et al. 2020). Additionally, genotypes IR58443-6B-10–3, IR16T1110, IR16T1086, IR16T1661, and IR16T1018 were also included in the breeding pool, and these genotypes have shown to have high performance under the direct-seeded conditions (DSR) along with salinity tolerance (IRRI personal communication). The elite pool consists of 8 genotypes from the freshly nominated 25 varieties for eight countries in 2021–2022. These genotypes revealed high salinity tolerance and superior grain yield, which provide ample evidence that conscious efforts are being made to develop the high-yielding lines under saline environments to achieve desired genetic gains.

Interestingly, the nominated variety IR63307-4B-4–3 for three countries, Indonesia, Vietnam, and Sri Lanka, has been bred by crossing IR 51511-B-B-34-B/TCCP 266–2-49-B-B-3 using a single cross-breeding strategy. The donor parent used here, TCCP 266–2-49-B-B-3, is a soma clonal variant of Pokkali with superior characteristics, including semi-dwarf plant type with white pericarp, medium consistency of grain type and possesses high yield potential along with vigorous growth without lodging. All eight superior breeding values harboring nominations genotypes, viz. IR121094-B-B-AJY3-2-B, IR121188-28–1-CMU2-2-B, IR117676-318–1-1–1, IR112462-B-25–2-1–1, IR16T1009, IR63307-4B-4–3, IR117839-22–15-B-CMU10-1-B, and IR117749-B-B-CMU6-1-B, are part of the elite panel. Conclusively, the selected lines for the elite pool development do not have only high breeding values for yield but possess a tolerance under salinity stress. The genotypes will be a great source of readily available variation to use in recurrent selection and further recombine and reshuffle to create an additional novel source of variation for grain yield and enhance the genetic gains.

## Conclusions

The current rate of genetic gains observed in the salinity breeding program is comparatively lower than the required genetic gain rates of 1.5% or above (Li et al. 2018). The rate of genetic gain in rice will increase to 2.5% or above in 2050 (Xu et al. 2017, 2020b). To deliver higher rates of genetic gains in the salinity breeding of IRRI, a holistic and systematic breeding effort with the integration of modern tools and technologies is required. A population improvement breeding strategy based on an elite x elite scheme with the integration of novel technologies like genomic selection, high-throughput phenotyping, and rapid recycling is highly required (Al-Tamimi et al. 2016; Li et al. 2018; Jighly et al. 2019; He and Li 2020). The elite breeding pool identified in this study would be the most potent and readily available genetic resource to drive the population improvement-based breeding strategy in IRRI’s salinity breeding program.

### Supplementary Information

Below is the link to the electronic supplementary material.

Supplementary file1 (XLSX 216 KB)

Supplementary file2 (DOCX 817 KB)

## Data Availability

The full set of historical trials along with pedigree information can be requested from the corresponding author. The R scripts used for the phenotypic data analysis and estimation of genetic trends from the historical trials are available at the GitHub repository and can be accessed through the following links: https://github.com/whussain2/Genetic_Trend_Rice_Drought https://github.com/whussain2/Analysis-pipeline
