# Supplementary material for: Genetic gains in IRRI’s rice salinity breeding and elite panel development as a future breeding resource
Source: Theor Appl Genet. 2024 Jan 31;137(2):37. doi: 10.1007/s00122-024-04545-9 (PMC10830834; doi:10.1007/s00122-024-04545-9)
Supplement: Supplementary file 2 — Supplementary file2 (DOCX 817 KB) [file 122_2024_4545_MOESM2_ESM.docx]

# Supplementary Information

**Rice Salinity Breeding at IRRI: Genetic Gains Achieved and Future Breeding Resource**

Apurva Khanna^1^, Joie Ramos^1^, Ma Teresa Sta. Cruz^1^, Margaret Catolos^1^, Mahender Anumalla^1^, Andres Godwin Sajise^1^, Glenn Gregorio^2^, Shalabh Dixit^1^, Jauhar Ali^1^, Md Rafiqul Islam^4^, Vikas Kumar Singh^4^, Akhlasur Rahman^3^, Hasina Khatun^3^, Daniel Joseph Pisano^1^, Sankalp Bhosale^1^, Waseem Hussain^1^*

^1^Rice Breeding Innovation Platform, International Rice Research Institute (IRRI), Los Baños, Laguna 4031, Philippines.

^2^Southeast Asian Regional Center for Graduate Study and Research in Agriculture (SEARCA) and University of Philippines, Los Baños, Laguna 4031, Philippines.

^3^Plant Breeding Division, Bangladesh Rice Research Institute (BRRI), Gazipur 1701, Bangladesh.

^4^IRRI South Asia Regional Center (IRRI-SA Hub), Hyderabad, Telangana 502324, India

**Supplementary Figures:**

**
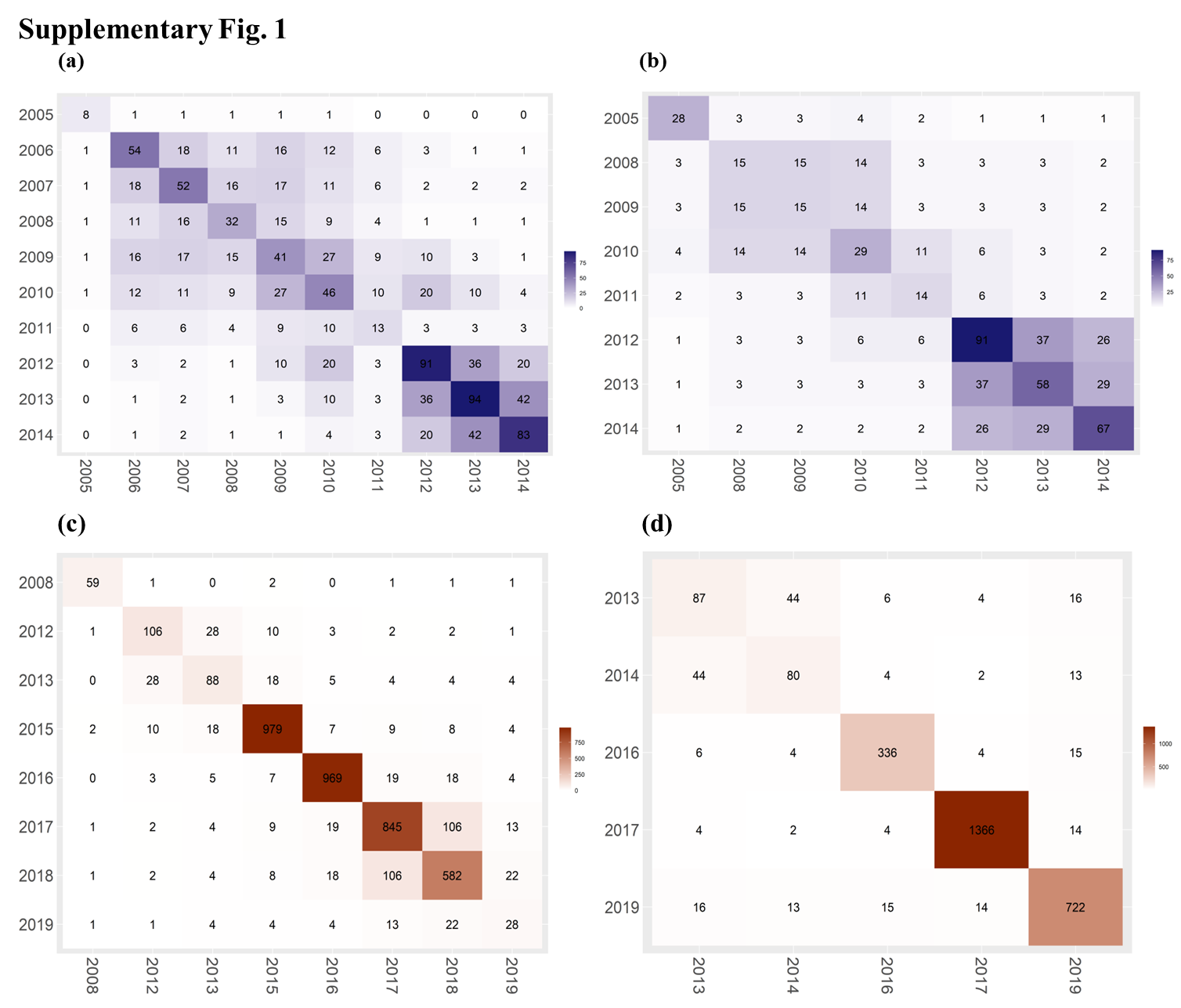
**

The figures depicted in brown depict the connectivity of the unique genotypes from Bangladesh evaluated during **(a)** Aman season, **(b)** Boro season; The heat maps plotted in blue depict the connectivity of the unique genotypes from the Philippines dataset tested during the **(c)** wet season, **(d)** dry season. The numbers in each box represent common genotypes between each year combination. Both datasets represent apt connectivity across years since the checks and promising varieties were repeatedly bred and tested in successive years to evaluate their performance.

**
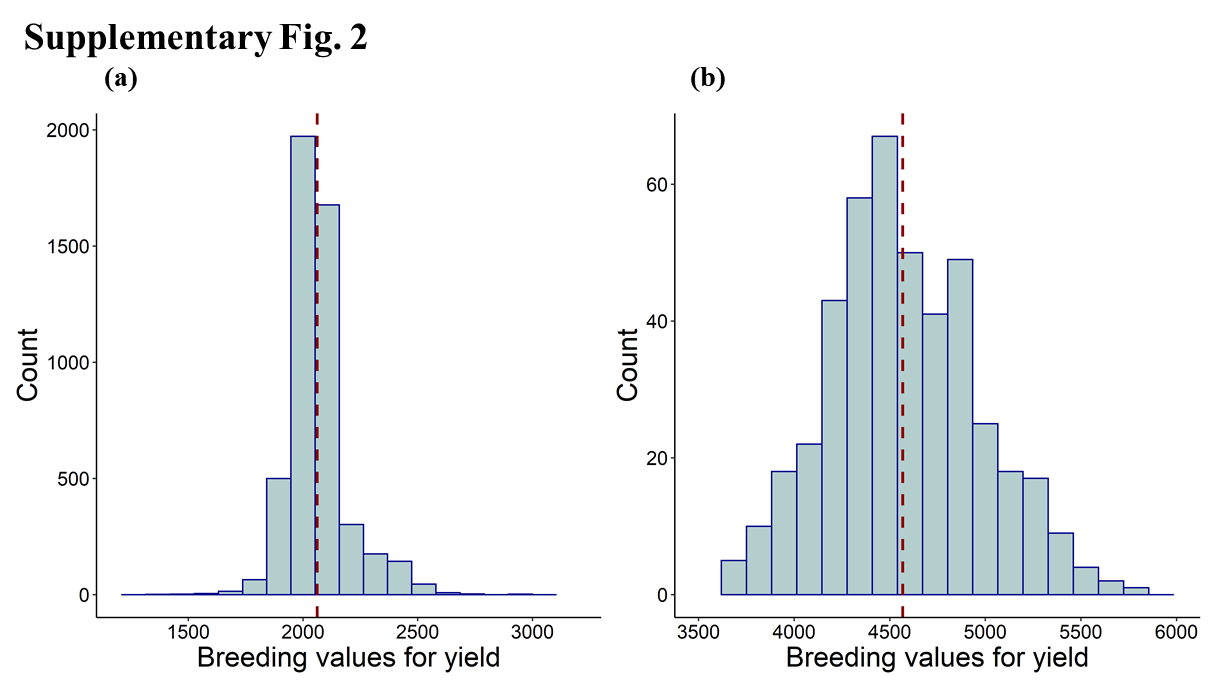
**

Supplementary Fig. 1

**3
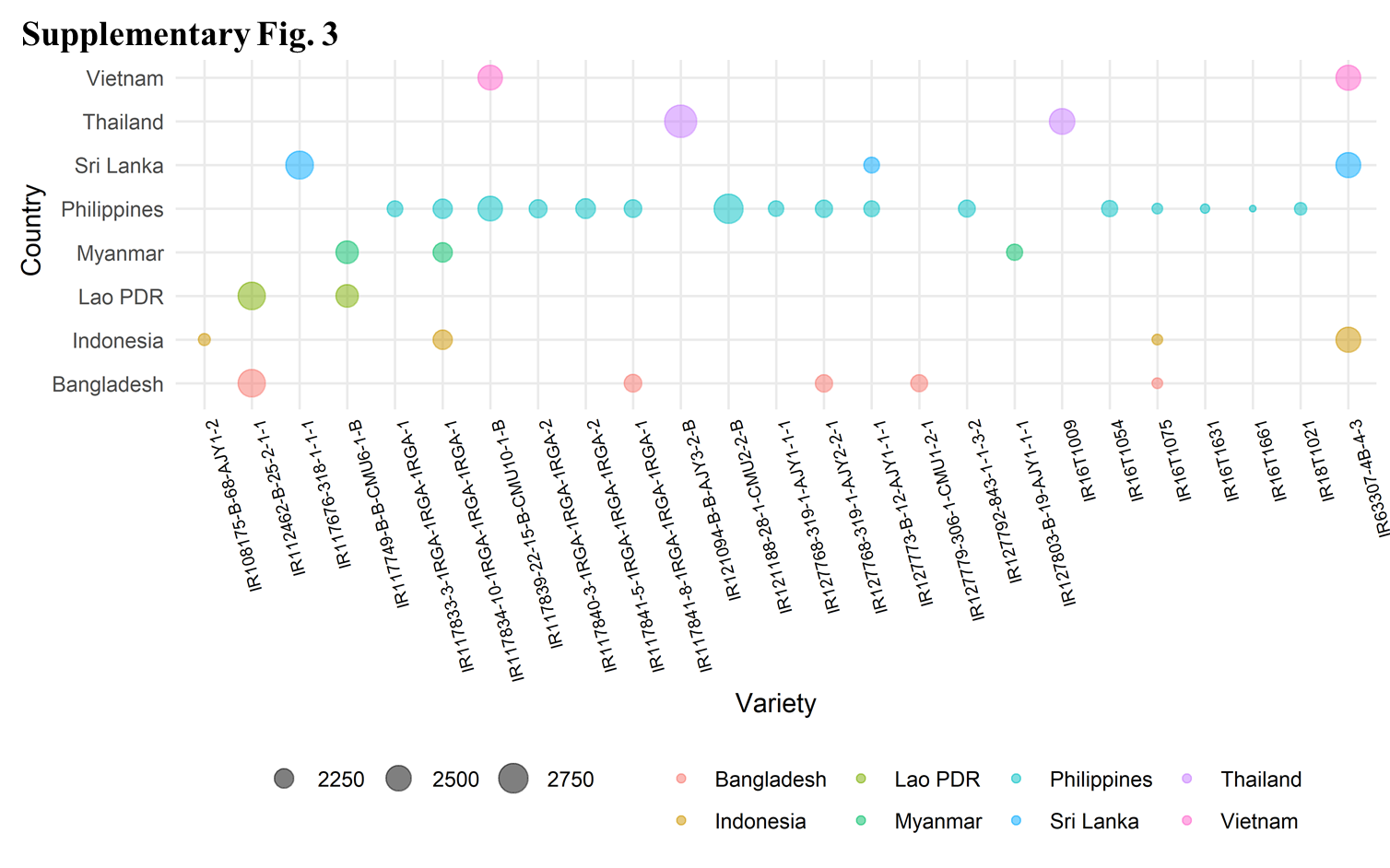
**

Salt tolerant historical breeding lines bred across 12 years from 2008-2019 at IRRI, Philippines and were selected for nominations across 8 countries for the year 2021-22. The breeding values for each of the countries have been depicted by different colors. The more the circumference of the circle, higher the breeding values. The highest breeding value was for the genotype IR121094-B-B-AJY3-2-B, nominated for Thailand depicted in violet color, followed by IR121188-28-1-CMU2-2-B, nominated for the Philippines depicted in cyan color.

**
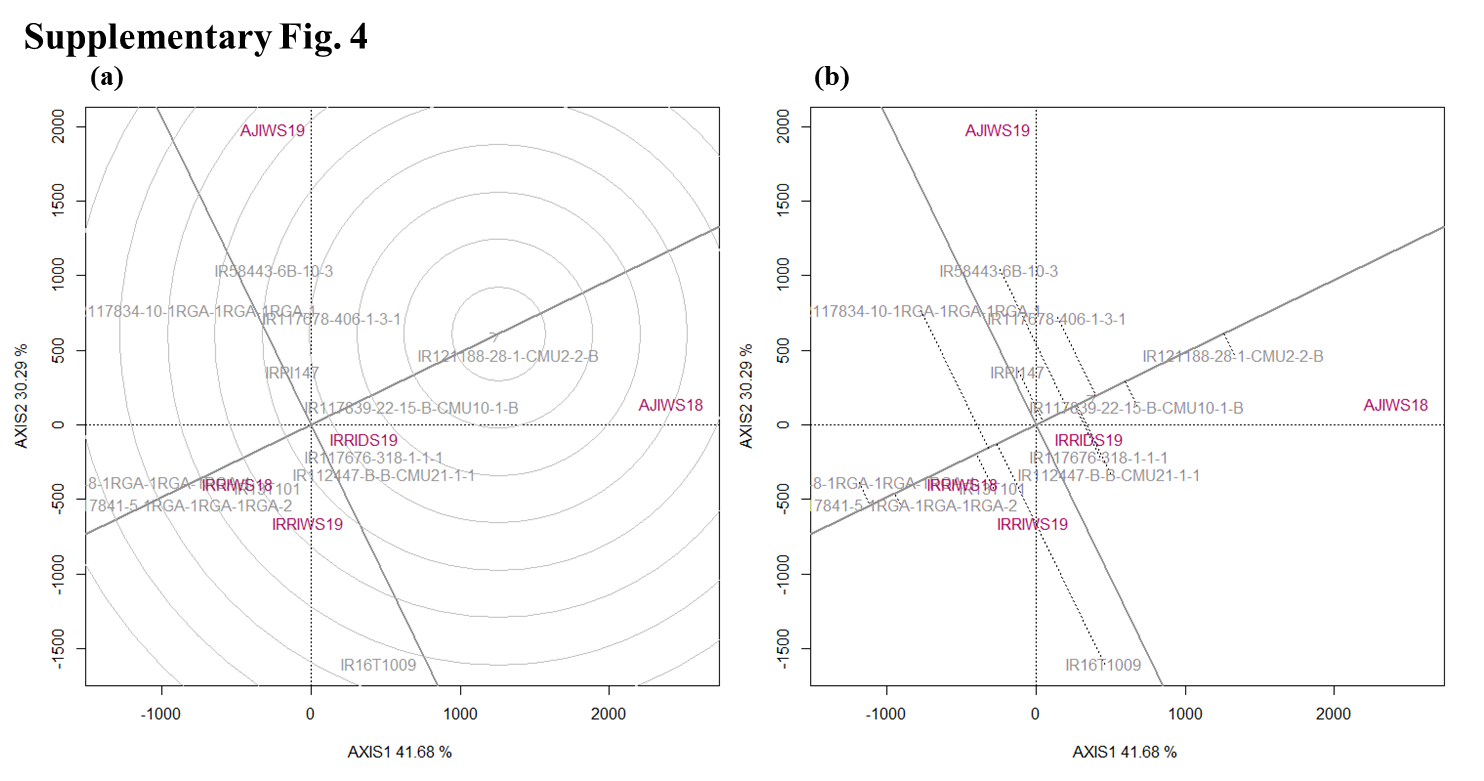
**

The goodness of fit of the biplot, explaining a total of 71.97% of test centric data (G+GE) has been represented in the figures. The percentages of GGE explained by the top two PC axes were estimated for ranking genotypes based on their relative performance and ranking genotypes relative to the ideal genotype. **(a)** Ranking of genotypes with reference to the “ideal genotype”, as shown by the arrow. The genotypes are raked in descending order starting from the arrow of ideal genotype. **(b)** Comparative depiction of genotypes based on their mean performance and stability portraying the average-environment coordination view of GGE biplot.
